# Factors Which Predispose Elderly Patients to Develop Peptic Ulcer Disease

**Published:** 1992-08

**Authors:** A. F. Safe

**Affiliations:** Senior Registrar, General and Geriatic Medicine Bristol Royal Infirmary, Bristol BS2 8HW


### Factors which predispose elderly patients to develop peptic ulcer disease

**Dr. A. F. Safe, M.Sc., M.R.C.P.**
Senior Registrar, General and Geriatic Medicine
Bristol Royal Infirmary, Bristol BS2 8HW.

### INTRODUCTION

Peptic ulcer disease (PUD) is am important cause of mortality and morbidity in the elderly. Despite a continuing diminution in the overall number of patients with PUD admitted to hospital, the proportion of patients over the age of 65 has steadily increased.[1] Several studies have shown that the incidence of gastric ulcer is higher,[2] its healing is slower[3] and the risk of relapse is greater in the elderly.[4] The incidence of gastritis without ulceration also arises dramatically with increasing age,[5] with some suggesting that up to 70% of patients over the age of 65 have evidence of gastritis.

### THE PATHOGENISIS OF PUD

PUD is multifactorial but a current view describes an imbalance between aggressive and the defensive factors.[6] Studying the effect of ageing on these factors is important in assessing why elderly patients are at high risk to develop PUD.

### AGGRESSIVE FACTORS

These include endogenous and exogenous factors.

Hydrogen ions and pepsin and bile refluxed from the duodenum are the main endogenous factors.

Most studies have concentrated on the role of the agrressive factors, particularly hydrochloric acid. This emphasis has tended to obscure the fact that acid alone has never been shown to induce an ulcer[7] and none of the measures used to control acid production can be said to cure ulcer disease permanently. Many studies have shown that basal and stimulated acid production diminish in the elderly[8] with a parallel increase in prevalence of achlorhydria and chronic gastritis with age in a parallel fashion.[9]

The pattern of change in pepsin secretion with age is less well defined. Using serum pepsinogen I as a marker of perpsin secretion, Samloff et al found that 40% of individuals over age 70 years had levels less than 50 µg/l, compared with only 5% of those under age 40.[10] There have been few studies of the amount of pepsin secreted in gastric juice. We have assessed the effect of ageing on the gastric aspirate acidity and peptic activity by comparing these biochemical parameters in the elderly and the young. The gastric aspirate acidity and peptic activity of elderly patients with gastritis and gastric ulcer was significantly lower than that of the young age group.[11]

Gastric bile reflux from the duodenum is common after gastric surgery. It causes a distinctive histological picture of reactive gastritis.[12] This can also occur in patients with a gastric ulcer who have not had surgery.[13] The frequency of spontaneous bile reflux in the elderly is not known.

### EXONGENOUS AGGRESSIVE FACTORS

Three exogenous factors emerges as those most clearly associated with ulcers and their complications; Helicobacter pylori (HP) infection, the use of non steroidal anti-inflammatory drugs (NSAIDS) and cigarette smoking. HP has been established as the most common and important cause of antral gastritis.[14] HP associated gastritis is present in 70–100% of patients with duodenal ulceration and 56–95% of those with gastric ulcers.[15] Several reports have confirmed the link between HP infection and the recurrence of PUD and have shown that eradication of the infection may cure the disease.[16] HP infection increases with age. In one study its prevalence was found to approach 75% in patients over 65 years of age.[17] O'Riordan and his colleagues found that HP infection has was associated with the majority of instances of symptomatic gastritis in the elderly.[18]

Treatment with non steroidal anti–inflammatory drugs (NSAIDS) is associated with an increased risk of PUD and its complications[19] and elderly patients are particularly vulnerable.[20] Several analyses have indicated a strong association between NSAIDS consumption and clinically serious PUD in elderly female patients.[21] The best overall approach to prevent NSAID mediated PUD is to reduce the frequency of NSAIDS use and exercise caution before prescribing these drugs in the elderly. Preventive management is hindered by the inability to define a particular high risk group and by difficulty in finding an acceptable and effective prophylactic treatment.

Smoking is associated with a high incidence of duodenal ulcers, to delay in healing and to increase in the relapse rate when drug therapy is stopped.[22] There are conflicting reports in the literature regarding smoking as a risk of gastric ulcer and its healing.[23]

### DEFENSIVE MECHANISMS

Mucosal resistance includes the mucus gel layer, a healthy functional epithelium and a good supply[24] and gastric mucus plays a key role.[24] Quantitative assessment of gastric mucus is difficult. One of the common methods is to analyse the quantity and spectrum of sugars from mucus glycoproteins in the gastric juice. Sialic acid is known to be an essential constituent of gastric mucus glycoprotein.[25] Recent studies showed that, with increasing age, gastric aspirate sialic acid levels fall significantly, indicating impaired mucus production.[11, 26]

### CONCLUSIONS

With increasing age, endogenous aggressive factors (gastric acidity and peptic activity) fall significantly, particularly in patients with gastritis and gastric ulcers, while defensive factors are significantly impaired. These changes would have important implications regarding peptic ulcer treatment in the elderly. HP infection is a very important pathogenic factor in PUD and, it is logical to consider eradication therapy for elderly patients with peptic ulcers associated with HP infection. NSAIDS induced gastric ulcers are common in patients with HP negative gastritis. Indentifying the subgroups of patients who are at high risk of developing NSAIDS gastropathy, will greatly help in detecting those who may benefit from prophylactic treatment.

### REFERENCES

1. Morris J, Dew M. (1985). Age and gastrointestinal disease. *In: Pathy MJ ed.* Principles and practice of geriatric medicine. London: John Wiley & Sons, **297-352**.
2. Sonnenberg A. (1988). Factors which influence the incidence and course of peptic ulcer. *Scand J Gastrenterol*, **23** (suppl 155) 119.
3. Piper D. W, Hunt J, Heap T. R. (1980). The healing rate of chronic gastric ulcer in patients admitted to hospital. *Scand J Gastroenterol*, **15**, 113-7.
4. Battaglia G, Di Mario F, Piccoli A, Vianello F, Farinati F, Naccarato R. (1987). Clinical markers of slow healing and relapsing gastric ulcer. *Gut.*, **28**, 210-5.
5. Siurala M, Iskoski M, Varis K *et al.* (1968). Prevalence of gastritis in a rural population: biopsy study of subjects studied at random. *Scand J Gastroenterol*, **3**, 211-23.
6. Tasman-Jones C. (1986). Pathogenesis of peptic ulcer disease and gastritis: importance of aggressive and cytoprotective factors. *Scand J Gastroenterol*, **21** (suppl 122) 1-5.

7. Mann F. C, Bollman J. L. (1932). Experimentally produced peptic ulcers. *JAMA*, **99**, 1576-82.
8. Grossman M. I, Kirsner J. B, Gillespie I. E. (1963). Basal and histalogstimulated gastric secretion in control subjects and in patients with peptic ulcer or gastric ulcer. *Gastroenterology*, **45**, 14-26.
9. Edwards F. C, Coghill N. F. (1966). Aetiological factors in chronic atrophic gastritis. *Br Med J.*, **2**, 1409-15.
10. Samloff I. M., Liebman W. K., Panitch N. M. (1975). Serum group I pepsinogens by radioimmunoassay in control subjects and patients with peptic ulcer. *Gastroenterology*, **69**, 83-90.
11. Safe A., Corfield A., Warren B., Mountford R. A. (1992). What factors predispose elderly stomachs to gastropathy? *Gut.*, **33**, (Suppl 1): S6.
12. Dewar E. P., Dixon M. F., Johnston D. (1984). Bile reflux and degree of gastritis in patients with gastric ulcer: before and after operation. *J Surg Res*, **37**, 277-84.
13. Thomas W. E. G. (1984). The possible role of duodenogastric reflux in the pathogenesis of both gastric and duodenal ulcers. *Scand J Gastroenterol*, **19**, (Suppl 92): 155.
14. Rauws E. A. J., Langenberg W., Houthoff H. J., Zanen H. C., Tytgat G. N. J. (1988). Camylobacter pyloridis - associated chronic active antral gastritis. *Gastroeterology*, **94**, 33-40.
15. O'Connor H. J., Dixon M. F., Wyatt J. I., Axon A. T. R. (1986). Campylobacter positive and Campylobacter - negative ulcers - have they a different aetiology? *Gut*, **27**, 1282A.
16. Marshall B. J., Goodwin C. S., Warren J. R. (1988). Prospective Double blind trial of duodenal ulcer relapse after eradication of campylobacter pylori. *Lancet*, **ii**, 1437-42.
17. Dwyer B., Kaldor J., Tee W., Raios K. (1989). The prevalence of Campylobacter pylori in human populations. In: *Rathbone BJ, Heatley RV, eds.* Campylobacter pylori and gastroduodenal disease. *Oxford: Blackwell Scientific publications*, **pl90**.
18. O'Riordan T. G., Tobin A., O'Morain C. (1991).Helicobacter pylori infection in elderly dyspeptic patients. *Age and ageing*, **20**, 189-92.
19. Sommerville K., Faulkner G., Langman M. (1986). Non-steroidal antiinflammatory drugs and bleeding peptic ulcer. *Lancet*, **1**, 462-64.
20. Armstrong C. P., Blower A. L. (1987). Non-steroidal anti-inflammatory drug and life threatening complications of peptic ulceration. *Gut.*, **28**, 527-32.
21. Jolobe O. M. P., Montogomery R. D. (1984). Changing clinical pattern of gastric ulcer. Are antiinflammatory drugs involved? *Digestion*, **29**, 164-170.
22. Sonnenberg A., Muller-Lissner S. A., Vogel E. *et al.* (1981). Predictors of duodenal ulcer healing and relapse. *Gastroenterology*, **81**, 1061-7
23. Okada M., Yao T., Fuchigami T., Imamura K., Omae T. (1984). Factors influencing the healing rate of gastric ulcer in hospitalised subjects. *Gut.*, **25**, 881-5.
24. Tasman-Jones C., Maher C., Thomsen L., Lee S. P., Vanderwee M. (1987). Mucosal defences and gastroduodenal disease. *Digestion*, **37**, (supp 2) , 1-7.
25. Jass J. R., Filipe M. I. (1981). The mucin profile of normal gastric mucosa, intestinal metaplasia and its variants and gastric carcinoma. *Histochem J*, **13**, 931-9.
26. Corfield A. P., Wagner S. A., Clamp J. R., Mountford R. A., Schauer R. (1986). Demonstration of 9-O-lactyl-N-acetylneuraminic acid in human gastric aspirates. *Biochem Soc Trans*, **15**, 391.

